# Supplementary material for: Laser-induced nitrogen fixation
Source: Nat Commun. 2023 Sep 13;14:5668. doi: 10.1038/s41467-023-41441-0 (PMC10499830; doi:10.1038/s41467-023-41441-0)
Supplement: Supplementary file 1 — Supplementary Information [file 41467_2023_41441_MOESM1_ESM.pdf]

Supplementary Information for

Laser-Induced Nitrogen Fixation

Huize Wang[1], Ranga Rohit Seemakurthi[2], Gao-Feng Chen[1,*], Volker Strauss[1], Oleksandr Savateev[1], Guangtong Hai[3], Liangxin Ding[4], Núria López[2, *], Haihui Wang[3,*], Markus Antonietti[1]

Supplementary Methods 2

Chemicals and materials 2

Detection of produced ammonia and performance evaluation 2

Density functional theory 3

Physicochemical characterization 4

Figure 1-5 4

Figure 6-10 10

Supplementary Note 1 13

Figure 11-15 14

Figure 16-20 16

Figure 21-25 18

Supplementary Note 2 22

Figure 26-30 23

Figure 31-37 25

Table 1-5 30

Table 6-10 33

Table 11-14 34

Supplementary References 35

Supplementary methods

Chemicals and materials

Lithium oxide (Sigma-Aldrich, 97%), magnesium oxide (Sigma-Aldrich, ≧99%), aluminum oxide (Sigma-Aldrich, Standard grade), calcium oxide (Sigma-Aldrich, 99.9%), zinc oxide (Sigma-Aldrich, 99.9%), hydrochloric acid (ACS, 37%), NaOH (Alfa, 98%), salicylic acid (ACS, ≧99%), sodium citrate (Sigma-Aldrich, ≧99%), NaClO (Honeywell, Reagent Grade, 10~15%), and Sodium nitroferricyanide(III) dihydrate $Na_2[Fe(CN)_5NO] \bullet 2H_2O$ (Sigma-Aldrich, ≧99%).

Detection of produced ammonia and performance evaluation

The generated ammonia was detected and quantified after the hydrolysis of Li_3N in 100 mL of 0.05 M hydrochloric acid solution. The generated ammonia from other metal nitrides was detected and quantified after the hydrolysis of metal nitrides in 15 mL of 0.05 M hydrochloric acid solution. The surface of the titanium sheet was refreshed, and the titanium nitride coating remains due to the high solubility of Li_3N in an acid solution. The ammonia in the 0.05 M hydrochloric acid solution was quantified by the indophenol blue method using the spectrophotometric analysis (fig S2) [1], whose maximum absorbance occurred at a wavelength of 660 nm. The ammonia yield rate is given by

$$\varepsilon = \frac{cV}{ts} \tag{1}$$

where c is the measured NH_3 concertation, V is the volume of the hydrochloric acid solution, t is the time of the laser-induced process and s is the working area of the laser in cm^{-2}. The correlated energy consumption is given by

$$E = \frac{pt}{cVM} \tag{2}$$

where p is the laser power used, t is the time of the laser-induced process, c is the measured NH_3 concertation, V is the volume of the hydrochloric acid solution and M is the relative molecular mass of NH_3. Considering the hydrolysis step is fast, in this work, only the laser-treated time was taken into account in calculating the yield rate.

For the isotope labeling experiment, $^{15}NH_3$ was quantified by ^1H NMR (400 MHz) with a recorded total of 1,000 transient scans. The spectra were collected in D_2O with maleic acid as

an internal standard. The scalar interaction between ^1H and ^{15}N in $^{15}NH_4^+$ results in a splitting of the ^1H resonance into two symmetric signals, with a spacing of 73.6 Hz.

Density functional theory

Density Functional Theory (DFT) simulations were performed using the Vienna Ab initio Simulation Package (VASP) [2-5] with the Perdew, Burke, and Ernzerhof (PBE) functional[6]. Further the DFT-D2 method [8] was used to include the van der Waals (vdW) corrections with our reparametrized C_6 coefficients for metals [9]. The core electrons were represented by the Projected Augmented Wave (PAW) pseudopotentials [7], while valence electrons were expanded in plane waves with a basis set cutoff energy of 450 eV. The converged lattice constants for the bulk fcc Li_2O and Li were 4.45 Å and 4.10 Å respectively, bcc MgO was 4.12 Å, while for the hexagonal Li_3N, they were a=3.47 Å, c=3.75 Å.

The Catkit package [10] was used to generate the Li_2O surfaces, with a (111) surface of 63 atoms and 6 layers thickness and a (211) surface of 120 atoms and 9 layers thickness. A Γ-centered k-point grid of 4x4x1 for (111) surface and 3x3x1 for (211) surface was employed which is generated through Monkhorst–Pack method with a reciprocal grid smaller than 0.03 $Å^{-1}$ [11]. All the simulations were spin polarized and a vacuum spacing of 15 Å was used in the z-direction. A dipole correction [12] was applied to remove the artifacts due to asymmetry in the z-direction. To determine the partial electron occupancies, the Gaussian smearing scheme was used with an energy smearing of 0.03 eV. The electronic step convergence was set to 10^{-4} eV. In general, the structures were converged till the forces were converged to within 0.03 eV/Å. In a few cases, to avoid restructuring of the surface, the stopping criterion for the simulations was set to an energy difference between two ionic steps of less than 10^{-3} eV.

For the estimation of the bulk formation energies Li_2O and Li_3N, the free energy of each species at 298 K was estimated using the following equation:

$$G = E_{DFT} + ZPE + \int C_P \, dT - TS \tag{3}$$

ZPE, Cp and S were estimated using the thermal properties tags in PHONOPY for the solids [13-14], while for the gases vaspkit [15] was used. The activation barriers were estimated using the Climbing Image Nudged Elastic Band (CI-NEB) method [16] developed by the Henkelman group. The initial six images between the initial and final state were generated by the Image Dependent Pair Potential (IDPP) method [17]. The phonon density of states for the bulk and the surface were generated using the PHONOPY package in combination with VASP [13-14]. All the simulations

have been uploaded to the iochem-BD database and can be accessed using the following link: https://doi.org/10.19061/iochem-bd-1-283.

Physicochemical characterization

Scanning electron microscopy was performed on a Zeiss LEO 1550 - Gemini system (acceleration voltage: 5 kV). EDX element maps were obtained at 10 kV acceleration voltage. X-ray diffraction measurements were performed on a Bruker D8 Advance diffractometer equipped with a scintillation counter detector with CuKα radiation (λ = 0.15418 nm) applying a 2θ angle in the range 5–80°. ^{1}H NMR spectra were recorded on Agilent 400 MHz. . The surface valence and chemical bonds of the samples were tested by X-ray photoelectron spectroscopy on an ESCALAB 250 spectrometer (Thermo Fisher Scientific) with monochromatic Al Kα radiation (1486.6 eV). The nitrogen content in synthesized TiN was measured using a LECO-TC500 ONH analyzer with a power of 5000 W (LECO Corporation, St. Joseph, MI). UV-vis absorption spectra were performed on a T70+ UV spectrometer (from PG instruments Ltd), which is a single split beam spectrophotometer available with a variable (0.5, 1, 2, 5nm) spectral bandwidth.

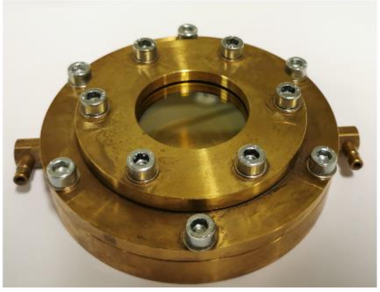

Supplementary Figure 1. Reaction cell: A photograph of a reaction cell for laser-induced nitrogen fixation.

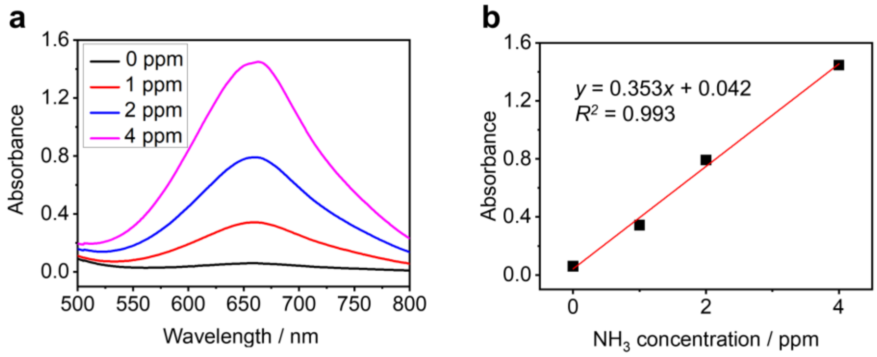

Supplementary Figure 2. Calibration for the Berthelot spectrophotometric analysis of ammonia: a. UV-vis absorption spectra of a solution prepared by mixing 2 mL of a defined amount of NH_3 in 0.05 M hydrochloric acid with 2 ml of a 1 M NaOH solution that contained salicylic acid and sodium citrate, 1 ml of 0.05 M NaClO, and 0.2 ml of 1 wt% $C_5FeN_6Na_2O$; **b.** Calibration curve using the data from panel (**a**). The ammonia was quantified by the indophenol blue method using the spectrophotometric analysis, whose maximum absorbance is observed at 660 nm.

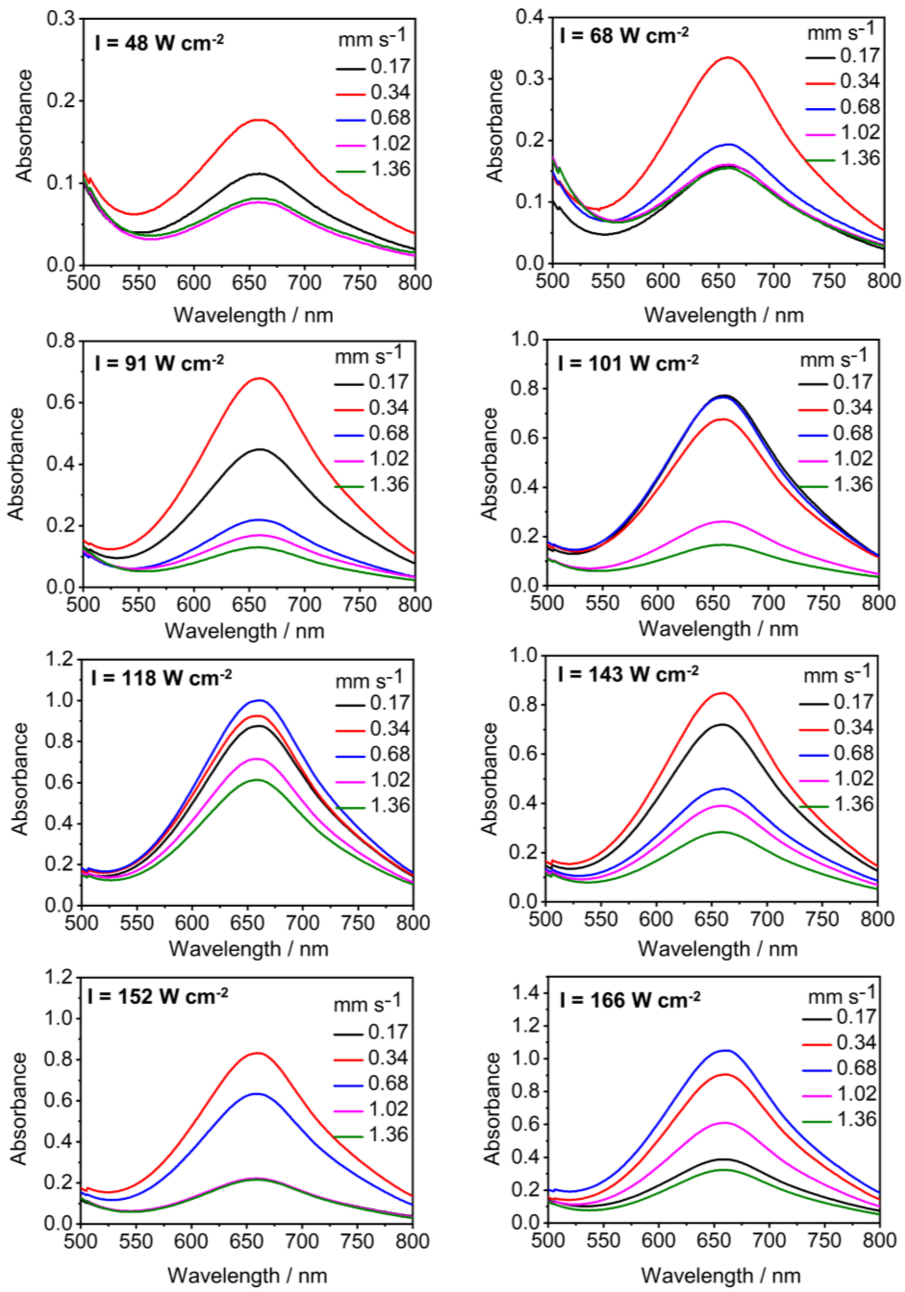

Supplementary Figure 3. UV-vis absorption spectra: for quantification of ammonia generated by hydrolysis of laser-induced Li_3N in dependence of laser power density (of 48, 68, 91, 101, 118, 143, 152, 166 W cm^{-2}) and scanning speed (of 0.17, 0.34, 0.68, 1.02, 1.36 mm s^{-1}) at N_2 pressure 7.5 bar.

Note: The primary role of laser irradiation is to facilitate the dissociation of metal-oxygen bonds, leading to the generation of zero-valent metals. The formation of nitrides, and subsequently the production of ammonia, is contingent upon both the reactivity of the metal and the reaction time (in this case, determined by the laser scanning speed). It is important to note that the dissociation of the metal-oxygen bonds necessitates a critical laser power density.

Once all the oxides have been induced and excited within the laser's range, further increasing the laser power density has minimal impact on promoting subsequent nitride formation.

In addition, the production yield of ammonia is influenced by several factors. These include the laser power density, which determines the energy required to break the metal-oxygen bond, the scanning speed of the laser, which affects the overall process time including metal oxide dissociation, and the reaction time of the activated zero-valent metal with nitrogen. Furthermore, the reactivity of the metal plays a crucial role in ammonia production. In essence, there exists an optimal scanning speed for a specific power density. Lower power densities necessitate longer reaction times, resulting in the higher ammonia yields. Conversely, higher power densities can achieve satisfactory results with shorter irradiation times, thereby consuming less energy and enhancing productivity. Therefore, multiple experimental parameters influencing each other lead to the results in Fig. S3: the slowest scanning speed (black, 0.17 mm s^{-1}) shows the least ammonia production at 68 and 152 W cm^{-2} but the best or second to the best at 48, 91, 101, and 143 W cm^{-2}.

;

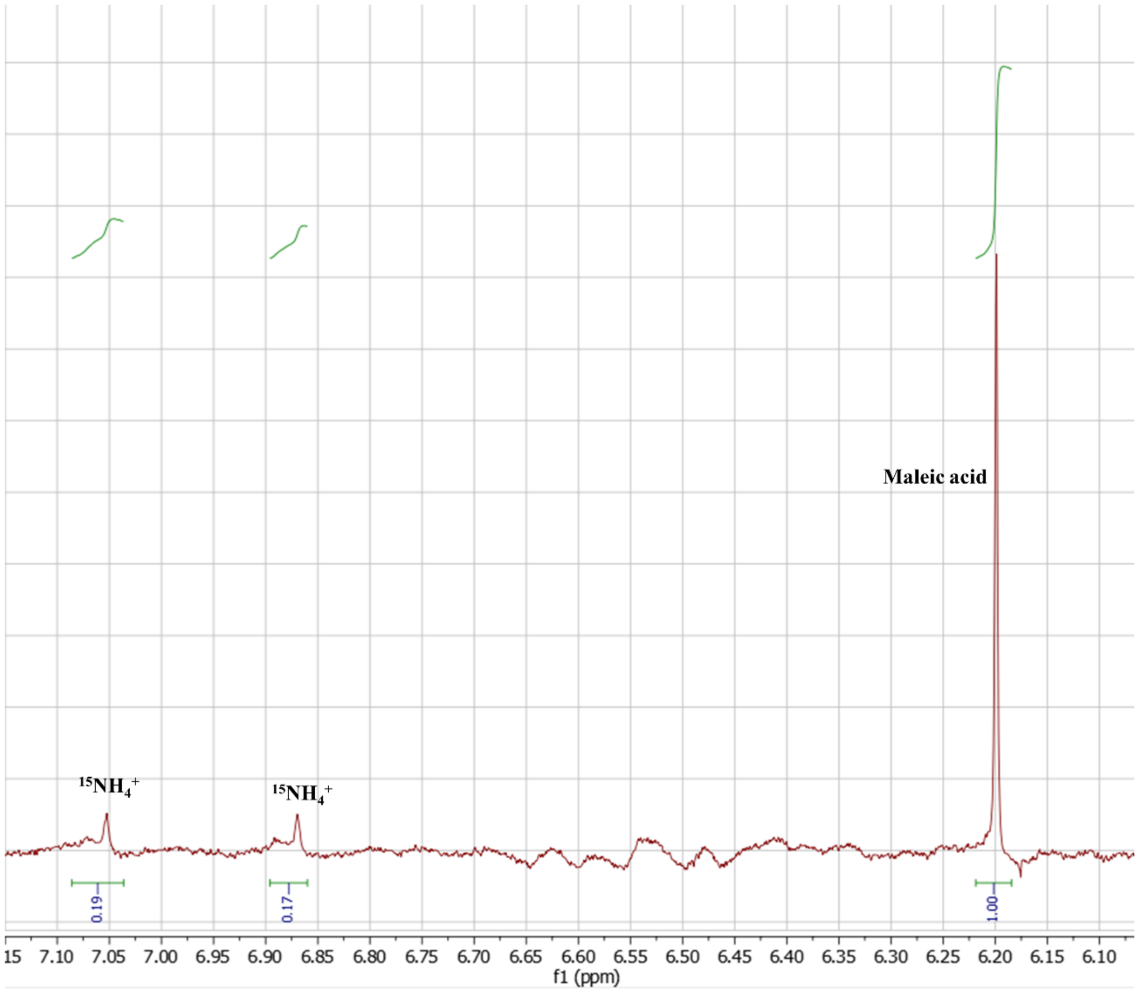

Supplementary Figure 4. ^1H NMR spectrum of the standard $^{15}NH_4Cl$ solutions with $^{15}NH_4^+$ concentration of 1 ppm.

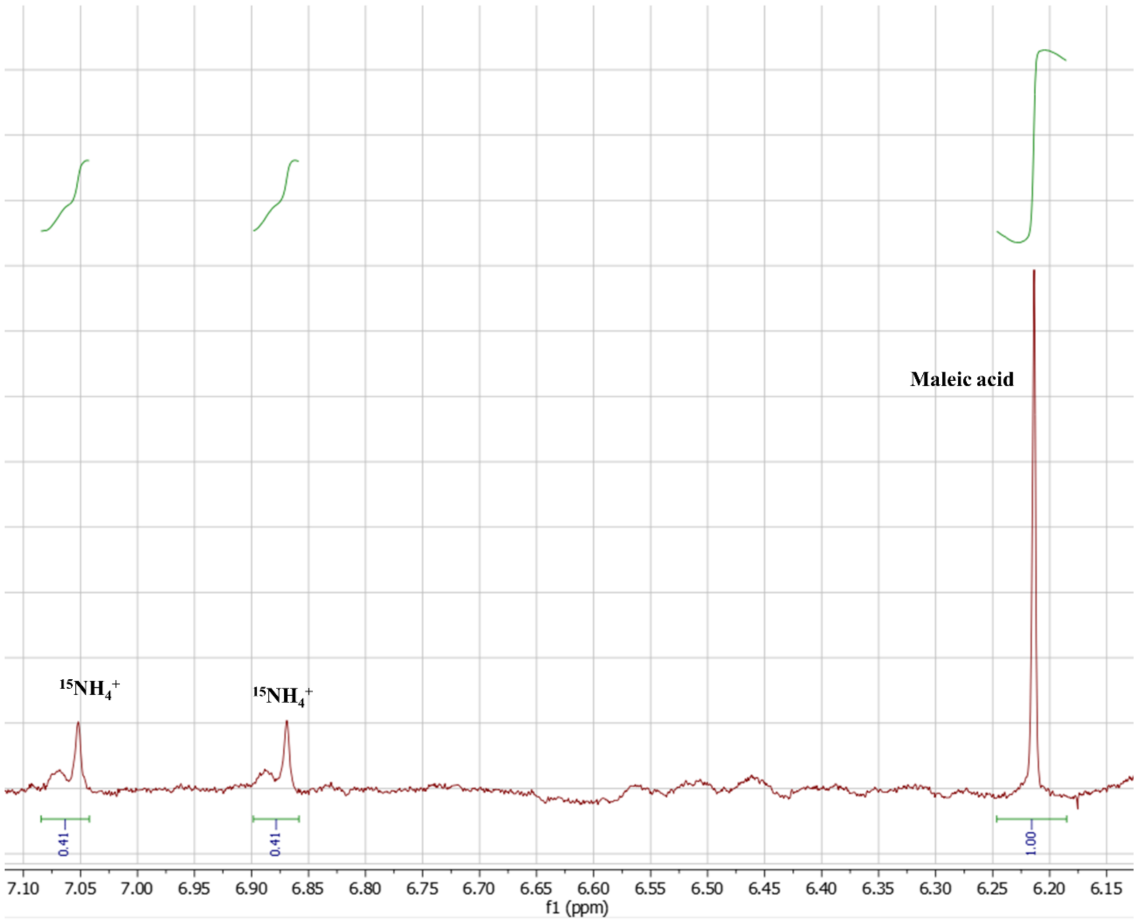

Supplementary Figure 5. ^{1}H NMR spectrum of the standard $^{15}NH_4Cl$ solutions with $^{15}NH_4^+$ concentration of 2 ppm.

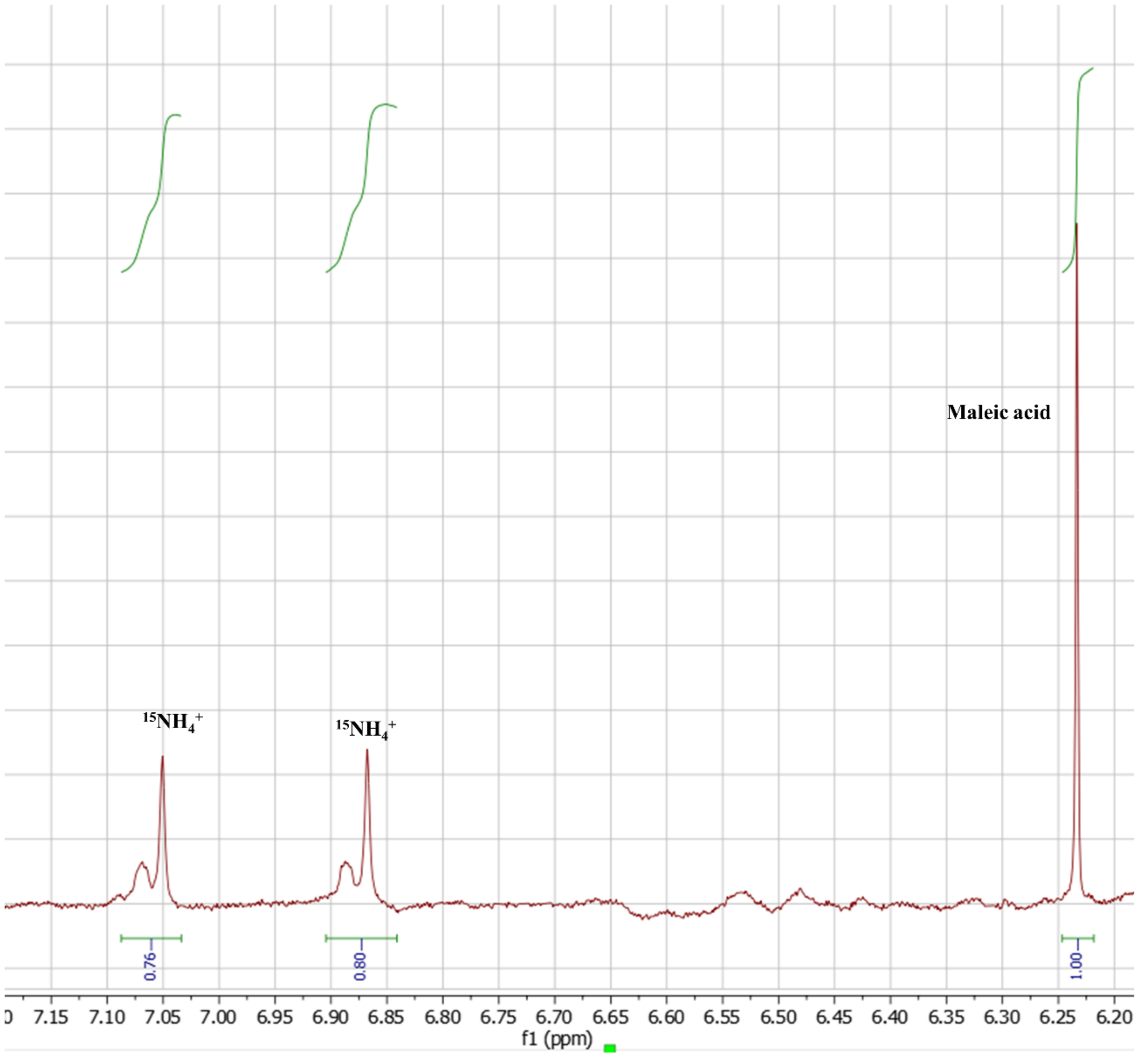

Supplementary Figure 6. ^{1}H NMR spectrum of the standard $^{15}NH_4Cl$ solutions with $^{15}NH_4^+$ concentration of 3 ppm.

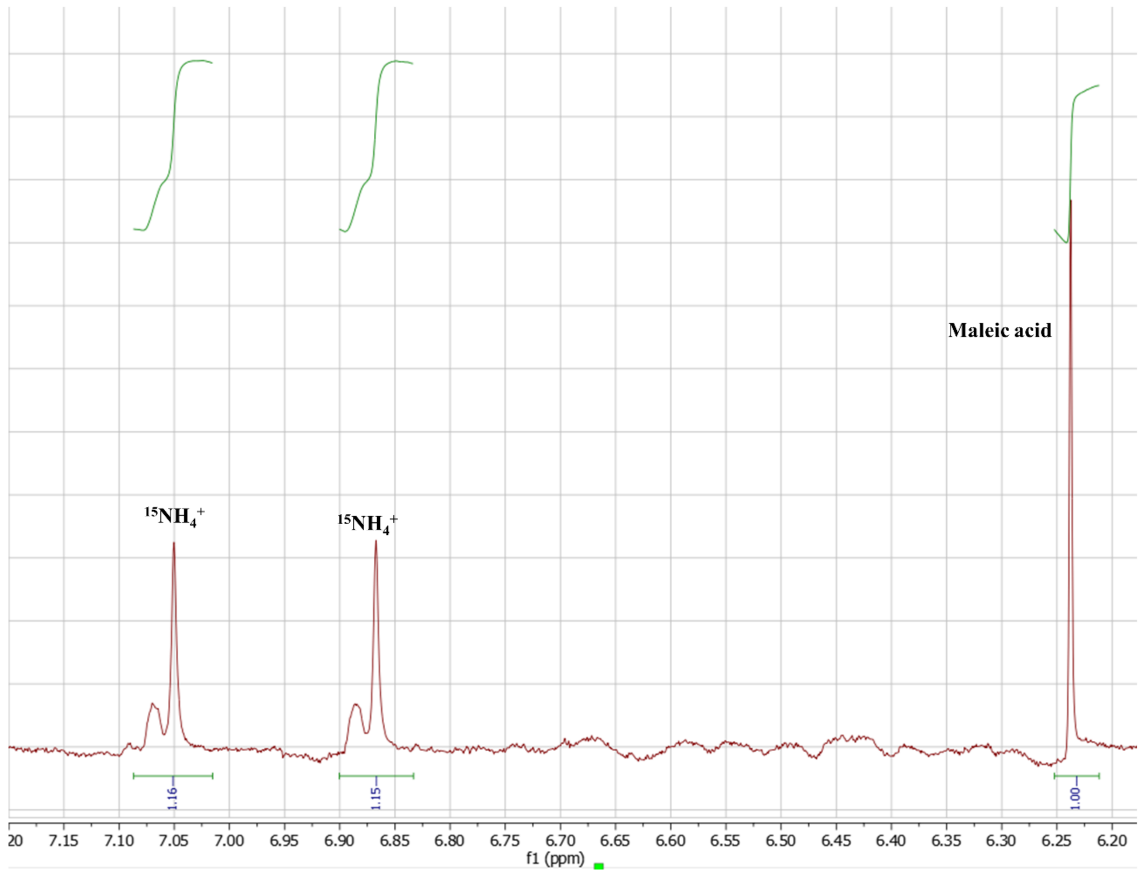

Supplementary Figure 7. ^1H NMR spectrum of the standard $^{15}NH_4Cl$ solutions with a $^{15}NH_4^+$ concentration of 4 ppm.

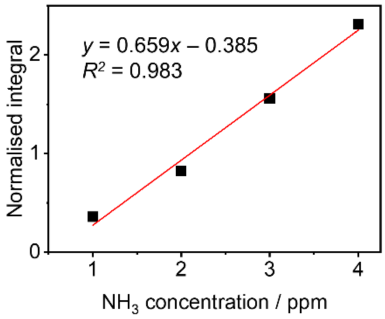

Supplementary Figure 8. ^1H NMR calibration plot for $^{15}NH_4^+$: normalised integral is obtained by dividing the integrated peak areas of $^{15}NH_4^+$ by the integrated peak area of maleic acid from the 1H NMR spectrum in Supplementary Figure 4-7.

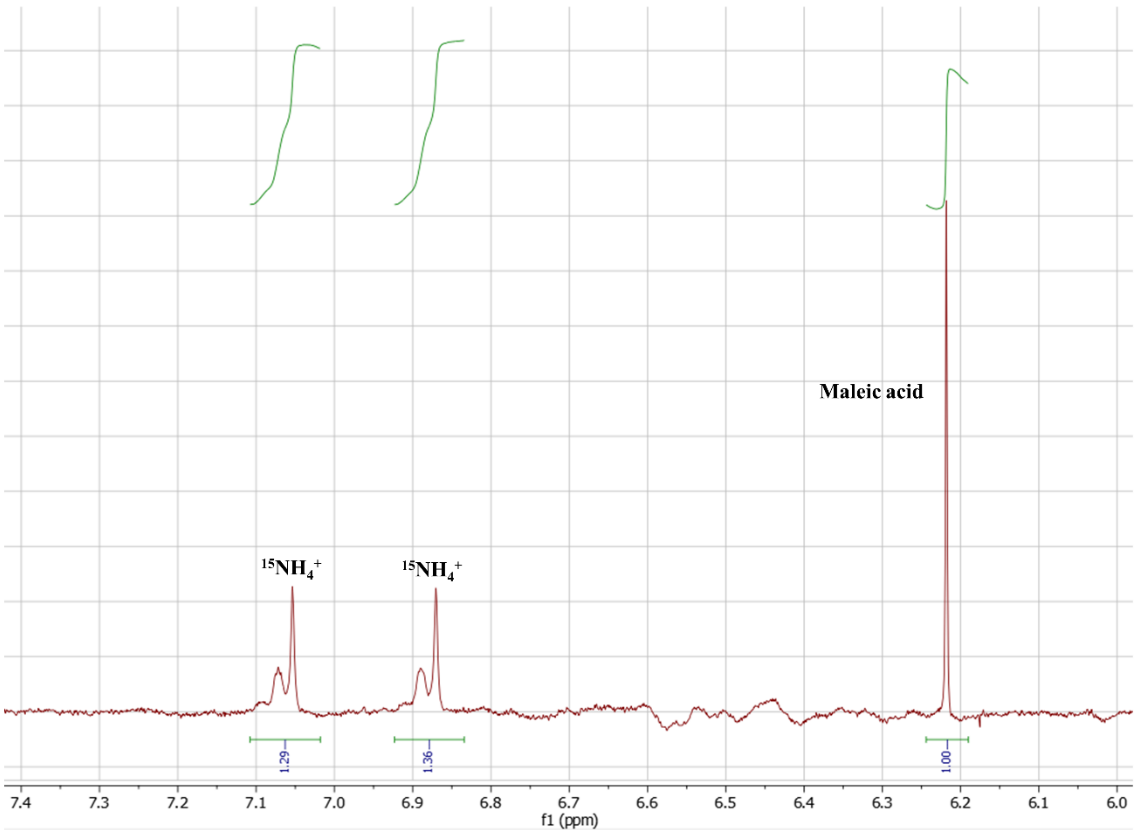

Supplementary Figure 9. ^{1}H NMR analysis for the Li_3N after hydrolysis: control experiments for the laser-induced process under $^{15}N_2$ atmospheres. ^{1}H NMR spectrum for NH_3 measurement after the nitridation and hydrolysis processes.

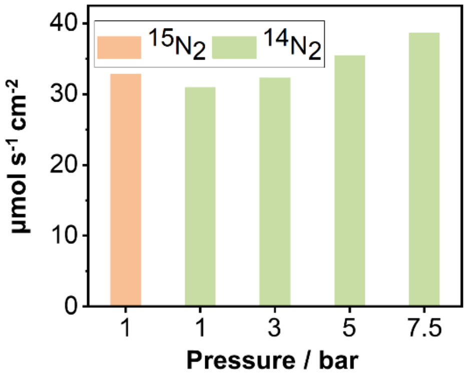

Supplementary Figure 10. Ammonia yield rate from hydrolysis of Li_3N synthesized under different $^{14}N_2$ pressure (1-7.5 bar) and 1 bar $^{15}N_2$: the ammonia yield rate increased slightly with increasing $^{15}N_2$ gas pressure. In addition, the ammonia yield rates under 1 bar $^{14}N_2$ and $^{15}N_2$ pressure are very close.

Supplementary Note 1

Our experimental results suggest a reaction pathway that the metal-oxide bond dissociates with the formation of metal, which then reacts spontaneously with nitrogen and forms metal nitride. In the Mg_{1s} region and Al_{2p} region of XPS spectra (fig. S17A), the peak signals attributed to zero-valent Mg and Al are detected, respectively. Besides, the EDX element maps of the laser-induced product obtained by using Al_2O_3 as a medium reveal a large amount of zero-valent Al – there is only a weak overlap between the map corresponding to this element and others (fig. S17B). In addition, zero-valent Al, Ca, and Zn (figs. S21, S23, and S24) were detected by SEM-EDX due to their less reactivity towards N_2 compared to zero-valent Li.

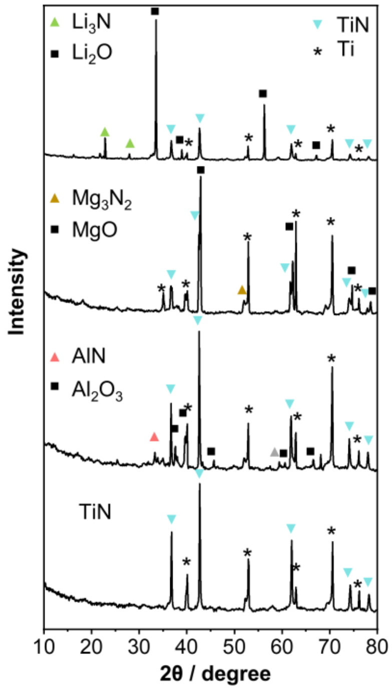

Supplementary Figure 11. XRD experiments: XRD patterns of laser-induced Li_3N, Mg_3N_2, AlN and TiN (after hydrolysis of Li_3N) films with the reference XRD patterns of: Li_3N (01-075-8959), Li_2O (01-086-3380), Mg_3N_2 (00-061-0096), MgO (04-004-7535), AlN (01-070-2545), Al_2O_3 (00-029-0063), TiN (98-000-0339), Ti (04-003-5042). In the LINF process, the Ti (substrate) also reacts with N_2 to form TiN, which takes place at a temperature above 1200 ^{o}C in the focused laser beam [18]. This TiN is very useful as it acts as a refractory passivation layer in further cycles.

Supplementary discussion for the *XPS* results:

In the C_{1s} region of all the laser-induced films, four peaks are detected at 284.8, 286.2, 285.5 and 289.9 eV, which are assigned to C-C, C-O, C=O and CO_3^{2-}, respectively [1]. The intensity of the peak assigned to CO_3^{2-} decreases sharply in the samples Mg_3N_2, AlN and TiN (after hydrolysis of Li_3N), which reflects the relatively low reactivity of Mg_3N_2, AlN and TiN toward carbon dioxide compared with lithium nitride.

Two peaks at 531.5 and 532.1 eV in the O_{1s} spectra are assigned to Li-O and Li-OH/Li-CO_3, respectively [19]. In the Li_{1s} spectrum, three peaks at 54.6, 55.1 and 55.6 eV are assigned to Li-N, Li-O, and Li-OH/Li-CO_3, respectively [19]. In the O_{1s} region of Mg_3N_2, three signals Mg-O, Mg-OH and contaminate (contam.) H_2O appear at 530.0, 531.5 and 532.5 eV [20]. Furthermore, two peaks at 530.8 and 532.1 eV are assigned to Al-O and Al-OH, respectively [21]. However, the high stability of titanium compounds is reflected in a prominent Ti-O peak and a minor Ti-OH peak at 530.4 and 532.0 eV, respectively [22].

For the Ti_{2p} XPS spectra of Li_3N and TiN, the detected peaks at 455.1,461.0, 456.4, 462.1, 458.5 and 464.3 are assigned to Ti-N ($2p_{3/2}$), TiN ($2p_{1/2}$), Ti-O-N ($2p_{3/2}$), Ti-O-N ($2p_{1/2}$), and TiO_2 ($2p_{3/2}$), and TiO_2 ($2p_{1/2}$). [23] Only in the XPS spectra of Mg_3N_2 and AlN, the peaks of exposed substrate titanium sheet are observed at 454.2 and 460.2 eV, which are assigned to Ti(0)($2p_{3/2}$), and Ti(0)($2p_{1/2}$).

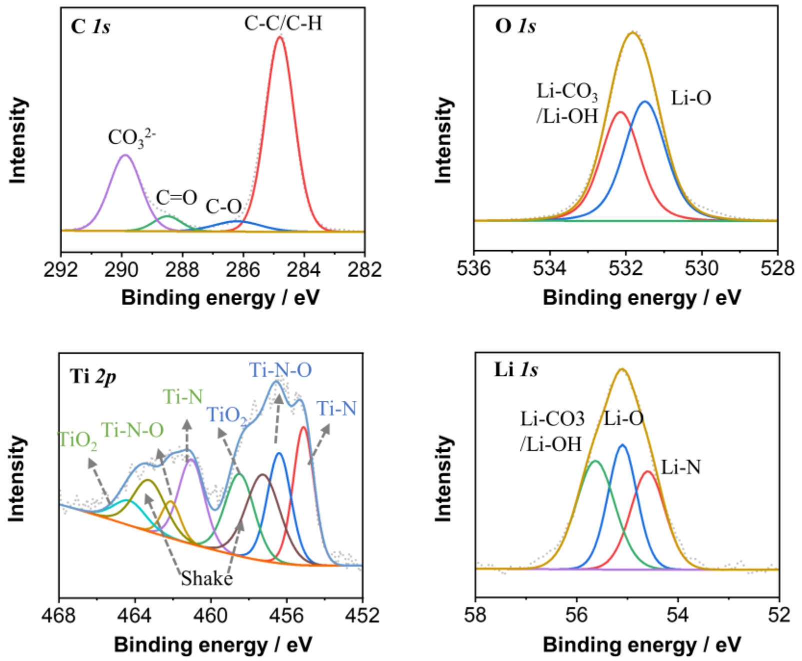

Supplementary Figure 12. *XPS* analysis of Li_3N film: *XPS* spectra of C_{1s}, O_{1s}, Ti_{2p} and Li_{1s} regions of laser-induced Li_3N film.

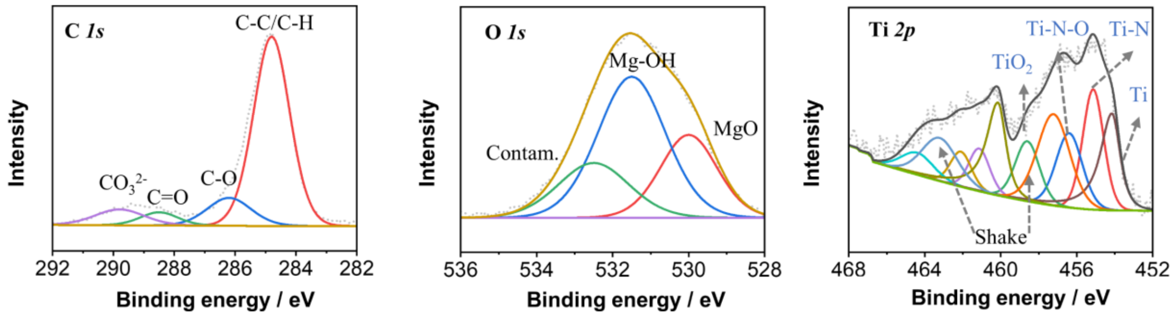

Supplementary Figure 13. ***XPS*** **analysis of Mg_3N_2 film:** *XPS* spectra of C*1s*, O*1s* and Ti*2p* regions of laser-induced Mg_3N_2 film.

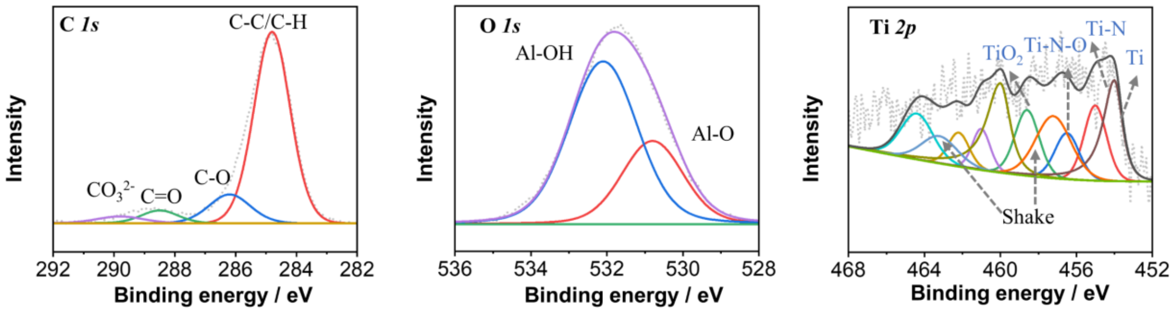

Supplementary Figure 14. ***XPS*** **analysis of AlN film**: *XPS* spectra of C*1s*, O*1s* and Ti*2p* regions of laser-induced AlN film.

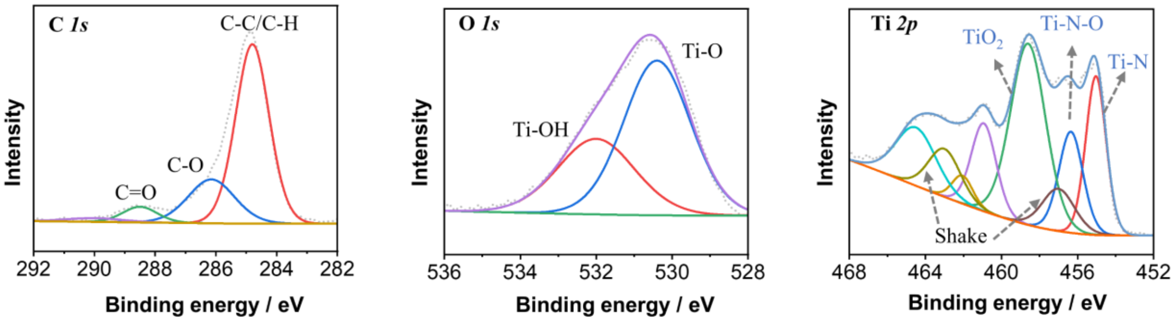

Supplementary Figure 15. ***XPS*** **analysis of TiN film:** *XPS* spectra of C*1s*, O*1s* and Ti*2p* regions of the laser-induced TiN film were exposed after the hydrolysis of Li_3N.

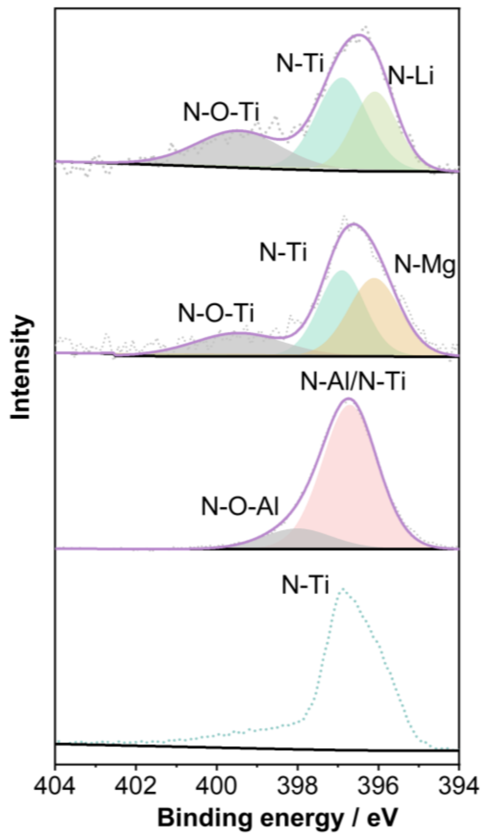

Supplementary Figure 16. Comparative *XPS* analysis of all films in N_{1s} region: N_{1s} region of *XPS* spectra of laser-induced Li_3N, Mg_3N_2, AlN and TiN (after hydrolysis of Li_3N) films. A strong N signal is observed on the surface of each laser-induced product. For the laser-induced products obtained by using Li_2O and MgO, the N_{1s} region displays three peaks at 396.1, 396.9 and 399.5 eV, which are assigned to N-Li [19], N-Mg [24], N-Ti and N-O-Ti [25], respectively. For the laser-induced product obtained by using Al_2O_3, the peaks at 396.7 and 398.0 eV are associated with N-Al/N-Ti and N-O-Al [26].

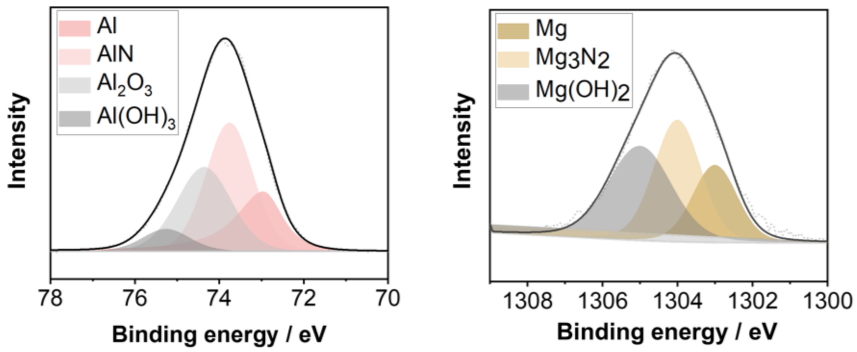

Supplementary Figure 17. Comparative *XPS* analysis of AlN and Mg_3N_2 films: *XPS* spectra of laser-induced AlN in Al_{2p} region and Mg_3N_2 in Mg_{1s} region: the Al_{2p} region displays four peaks at 73.0, 73.7, 74.3 and 75.2 eV are assigned to Al (0), AlN, Al_2O_3 and $Al(OH)_3$, respectively [21,27]. Mg_{1s} region displays three peaks at 1303.0, 1304.0 and 1305.0 eV are assigned to Mg(0), Mg_3N_2 and $Mg(OH)_2$, respectively [24].

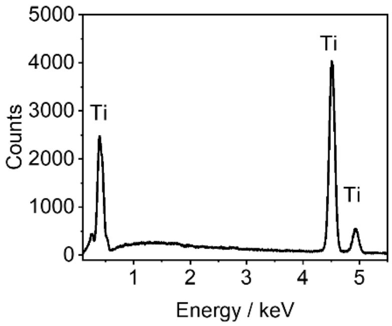

Supplementary Figure 18. EDX spectra of Titanium sheet

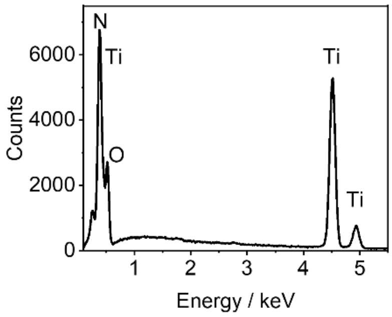

Supplementary Figure 19. EDX spectra of laser-induced Li_3N film

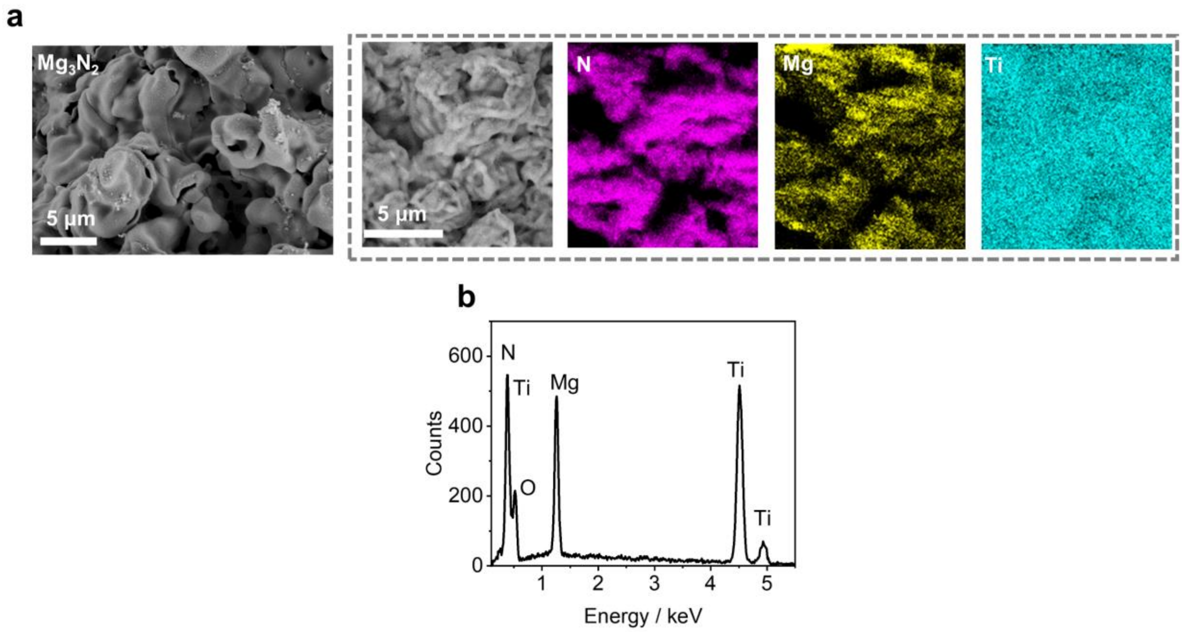

Supplementary Figure 20. SEM and EDX analysis of Mg_3N_2 film: a. Top-view of SEM micrograph of laser-induced Mg_3N_2 film with correlated EDX element maps; b. EDX spectra of Mg_3N_2 film.

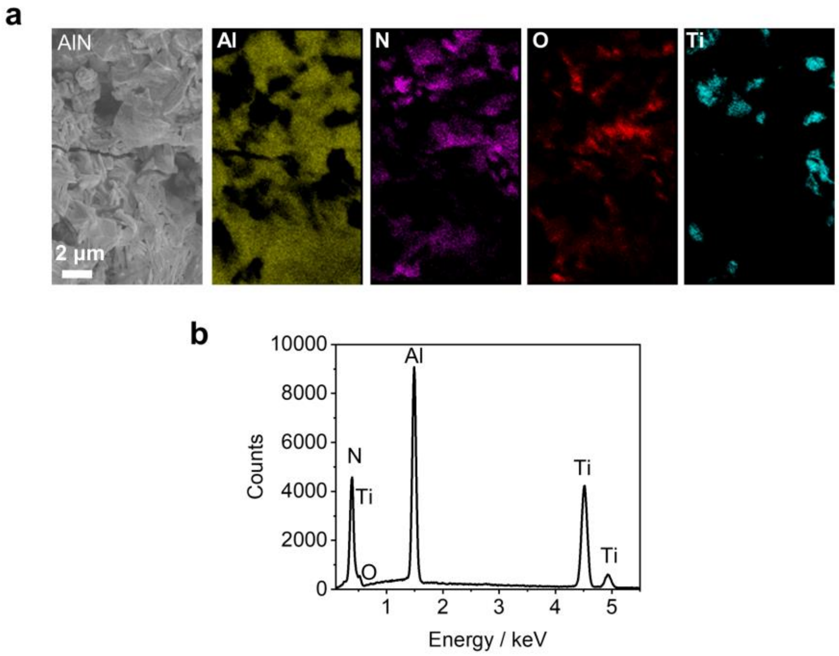

Supplementary Figure 21. SEM and EDX analysis of AlN film: a**.** Top-view SEM micrograph of AlN film with correlated EDX element maps; b. EDX spectra of laser-induced AlN film.

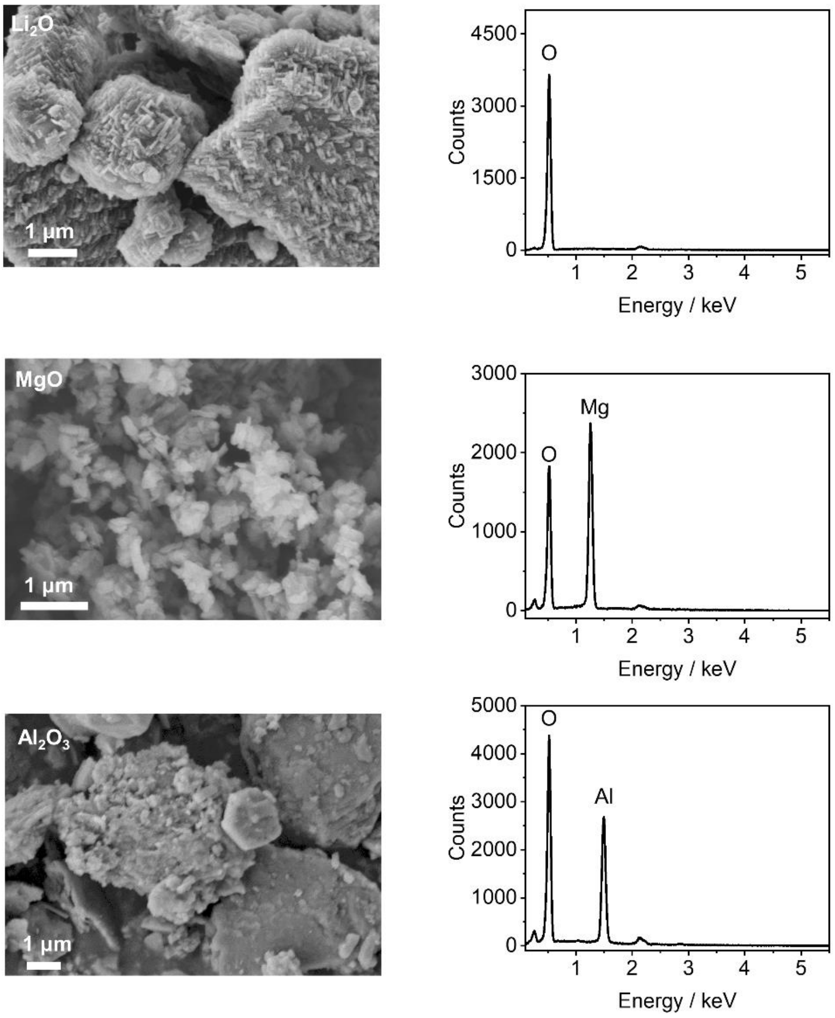

Supplementary Figure 22. SEM and EDX analysis of oxide precursor samples: SEM micrograph of Li_2O, MgO and Al_2O_3 powders with correlated EDX spectra: no nitrogen signal observed that indicates without impurities from nitrogen source.

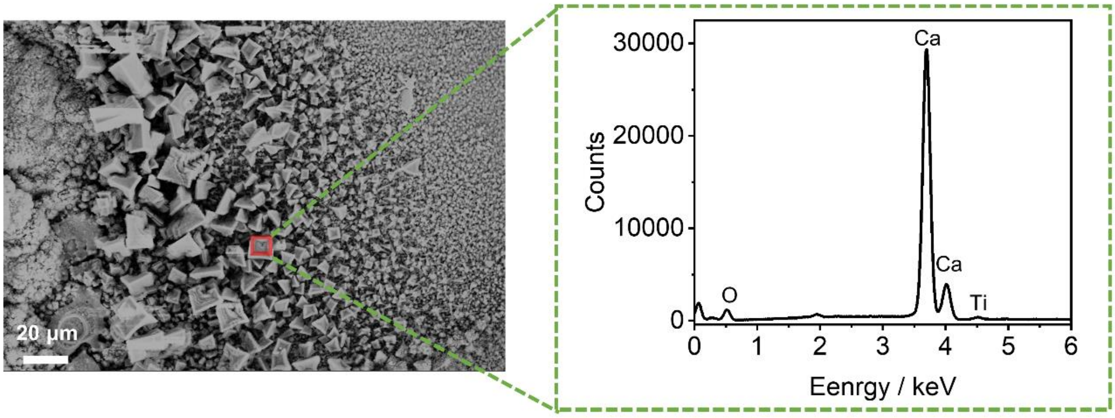

Supplementary Figure 23. SEM and EDX analysis of Ca_3N_2 film: top-view of the SEM micrographs of local Ca regions on the laser-induced Ca_3N_2 film with correlated EDX spectra.

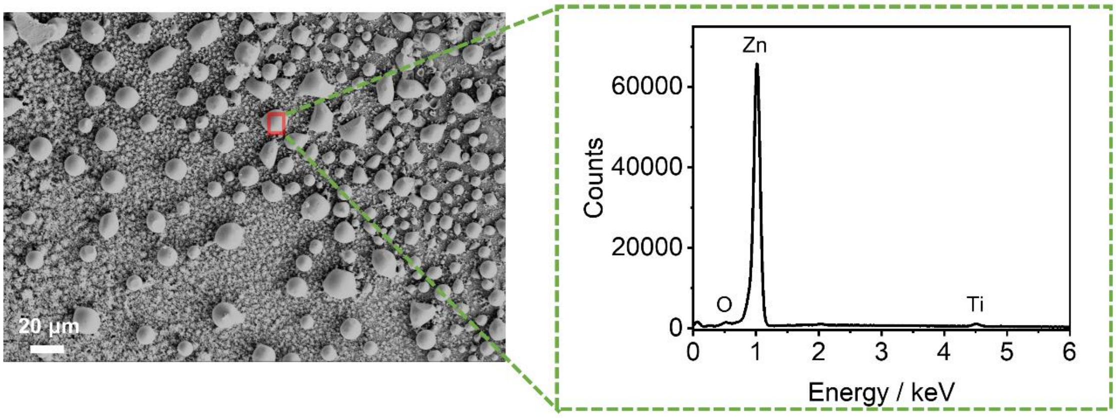

Supplementary Figure 24. SEM and EDX analysis of Zn_3N_2 film: Top-view of the SEM micrographs of local Zn regions on the laser-induced Zn_3N_2 film with correlated EDX spectra.

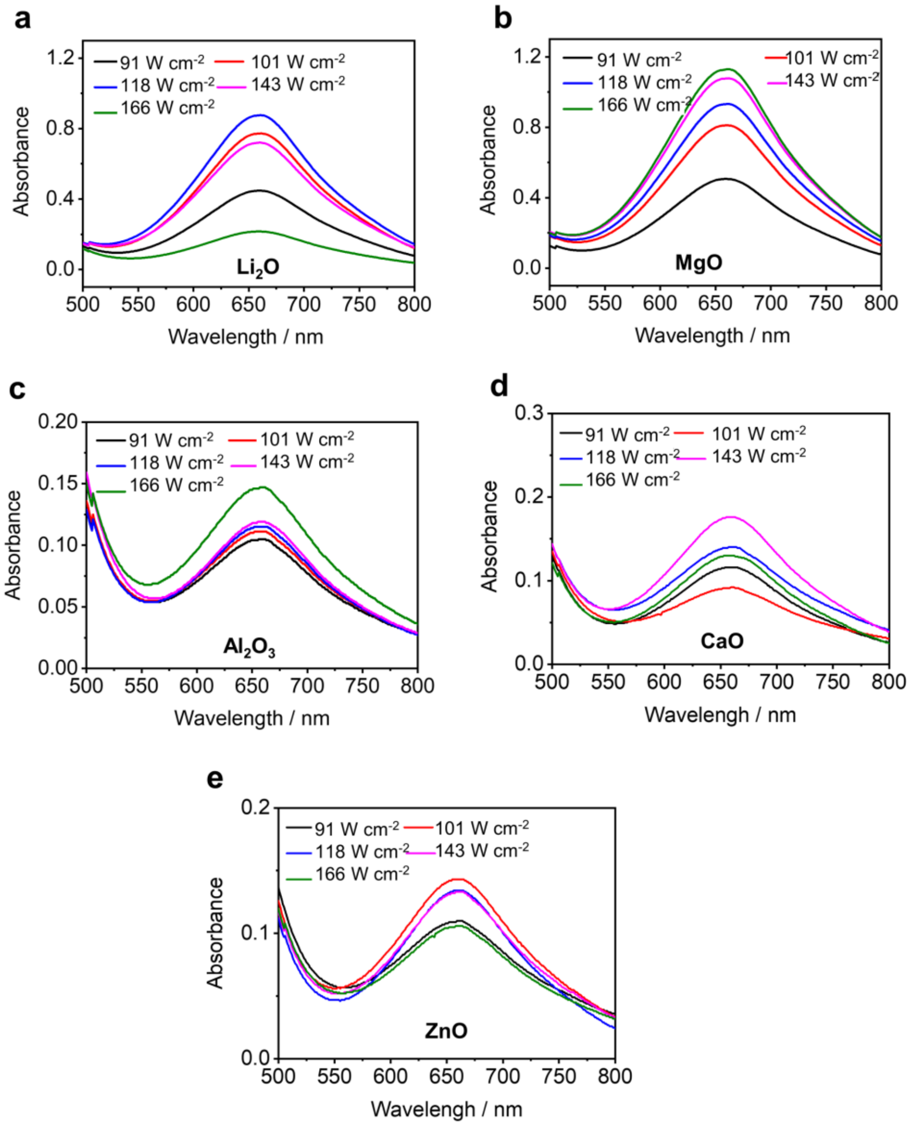

Supplementary Figure 25. UV-vis absorption spectra for quantification of ammonia: the ammonia generated by hydrolysis of laser-induced metal nitride from (a) Li_2O, (b) MgO, (c) Al_2O_3, (d) CaO and (e) ZnO. It is worth noting that the generated ammonia was detected and quantified after the hydrolysis of Li_3N in 100 mL of 0.05 M hydrochloric acid solution. The generated ammonia from other metal nitrides was detected and quantified after the hydrolysis of metal nitrides in 15 mL of 0.05 M hydrochloric acid solution.

Supplementary Note 2:

Li_2O (111) surface

For the terrace surface, among the three defects (Figure S28), the anti-site O-Li exchange is found to be energetically unfavourable by at least 2.04 eV compared to the Li adatom and peroxide formation for the terrace surface (Tables S4-6). For the Li adatom defect, Li^+ first migrates to the surface as an adatom with a reaction energy ΔE=1.82 eV (Table S4) and an activation energy ΔE_{act}=2.73 eV. To convert this adatom to metallic Li (0), an additional 3.20 eV is required. These surface Li (0) atoms can then activate N_2 and exothermically adsorb it (ΔE= -0.80 eV). On the other hand, for the peroxide defect, the oxygens in the surface combine to form a peroxide with an O-O distance of 1.525 Å (Table S7), leaving a vacancy in the lattice; this pathway is highly endothermic (ΔE= 6.65 eV). However, N_2 adsorption in this vacancy is strongly exothermic (ΔE= -1.98 eV), with the N-N bond distance increasing from the gas phase value of 1.115 to 1.294 Å.

Li_2O (211) surface

As mentioned in the main text, for the step surface among the three defects Li adatom (Figure S26) defect has the lowest energetics with the adatom formation requiring a reaction energy ΔE = 1.25 eV (Tables S4-6). To convert this adatom to meta-stable zero-valent species requires a reaction energy of 2.50 eV. In contrast, the peroxide defect formation is highly endothermic with a reaction energy ΔE = 6.28 eV. However, the adsorption of N_2 in the vicinity of the peroxide defect is strongly exothermic (ΔE = -1.77 eV), resulting in a reduced total reaction energy of 4.51 eV, which is 0.16 eV lower than the corresponding peroxide defect on the terrace surface (Table S5).

On the other hand, the anti-site defect formation which involves the exchange of lithium and oxygen atoms on the surface is highly endothermic (ΔE = 5.42 eV). Like the peroxide defect, the N_2 activation in the presence of the anti-site defect is strongly exothermic (ΔE = -1.48 eV). Importantly, the N-N bond distances increase to 1.270 Å during this process. The overall reaction energy for the anti-site defect is 3.94 eV, which is 2.77 eV lower than the corresponding defect on the terrace surface (Table S6). In all cases, the formation energies of defects were found to be lower on the Li_2O step surface than the terrace, consistent with the lower coordination numbers on step surfaces.

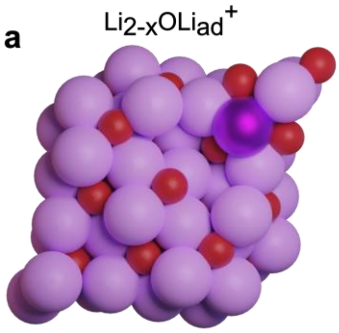

Supplementary Figure 26. Structures of defects in Li_2O (211) surface: (a) Adatom (Li_{ad}) defect, (b) Peroxide defect (O_{2v}), (c) Anti-site defect, where lithium and oxygen atoms are swapped with each other. Lithium and oxygen atoms involved in the defect are shown in dark purple and orange color respectively.

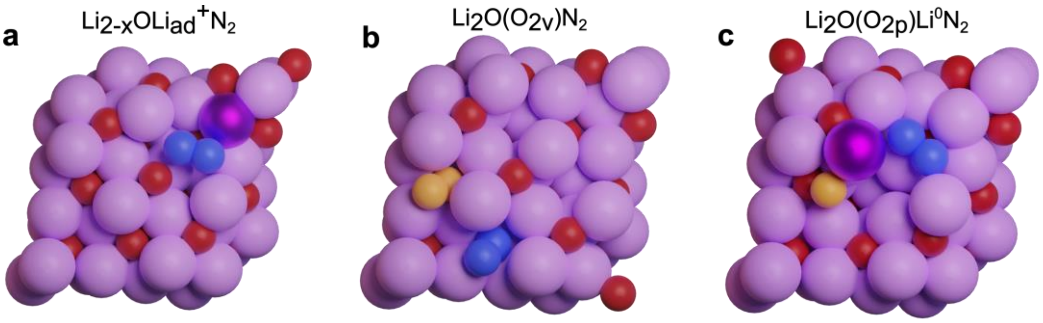

Supplementary Figure 27. N_2 adsorption in the Li_2O (211) surface defects: (a)Adatom (Li_{ad}) defect (b) Peroxide defect (O_{2v}), (c)Anti-site defect. Lithium and oxygen atoms involved in the defect are shown in dark purple and orange color respectively.

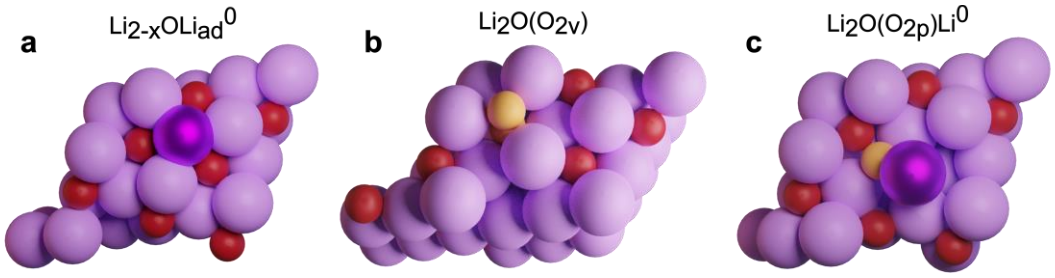

Supplementary Figure 28. Structures of defects in Li_2O (111) surface: (a) Adatom (Li_{ad}) defect, (b) Peroxide defect (O_{2v}), (c) Anti-site defect, where lithium and oxygen atoms are swapped with each other. Lithium and oxygen atoms involved in the defect are shown in dark purple and orange color respectively.

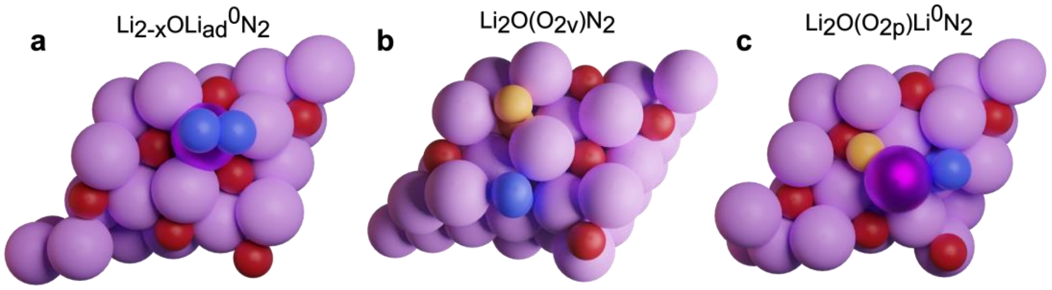

Supplementary Figure 29. N_2 adsorption in the Li_2O (111) surface defects: (a) Adatom (Li_{ad}) defect, (b) Peroxide defect (O_{2v}) (c) Anti-site defect. Lithium and oxygen atoms involved in the defect are shown in dark purple and orange color respectively.

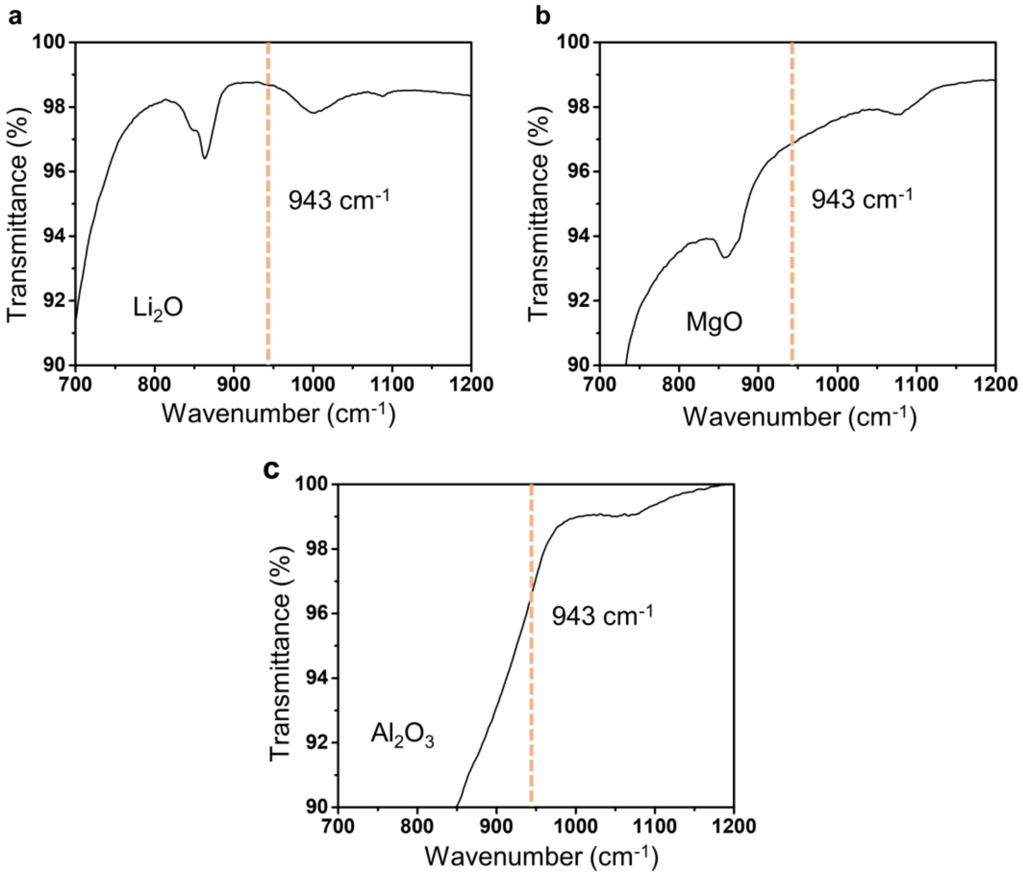

Supplementary Figure 30. Infrared Spectroscopy analysis: infrared absorption spectra of (a) Li_2O, (b) MgO, and (c) Al_2O_3 powders.

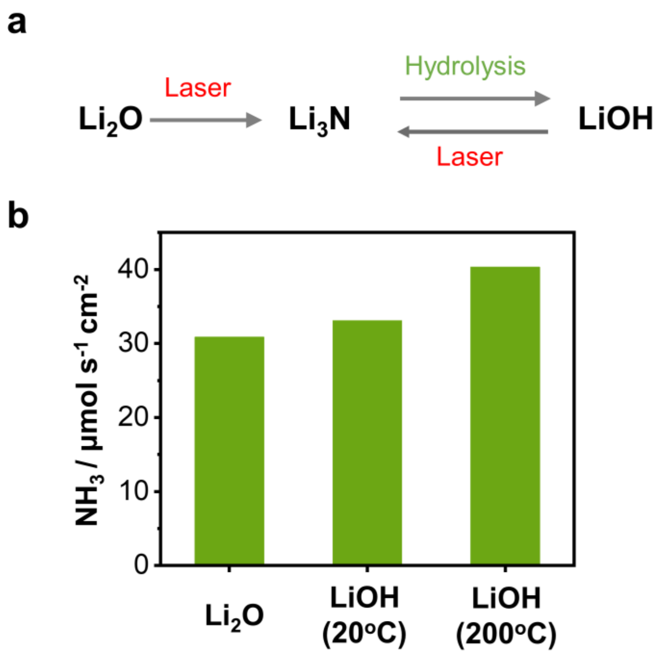

Supplementary Figure 31. Lithium cycle experiment by LINF process: a). Lithium cycling schematic diagram of the LING method by using laser power of 152 kW cm^{-2} and scanning speed of 2.73 mm s^{-1} under ambient pressure; b). Ammonia yield rate by using lithium oxide and lithium hydroxide, where the lithium hydroxide is generated through the hydrolysis of lithium nitride. Ammonia yields of relatively high magnitude were observed at 200°C. In the laser-induced process, the presence of moisture, subsequent to lithium hydroxide dehydration, can influence the formation of lithium nitride. An increase in temperature can aid in the removal of moisture from the reaction interface.

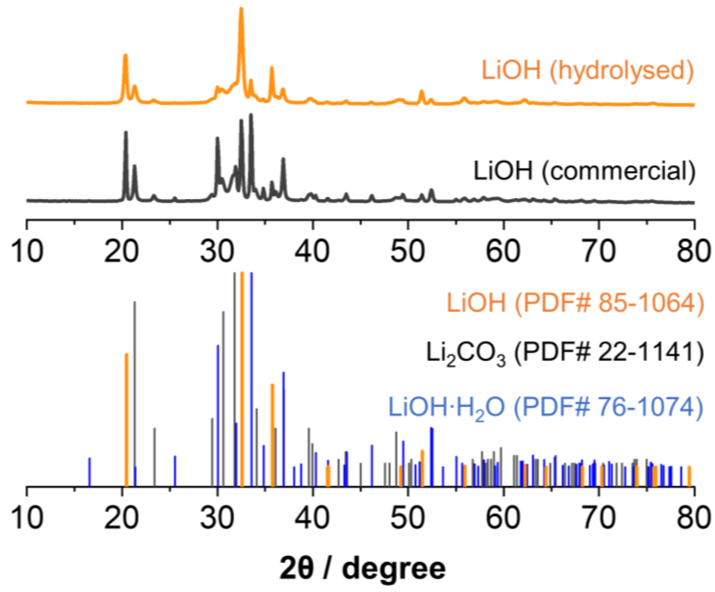

Supplementary Figure 32. Comparison of LiOH after Li_3N hydrolysis and commercial LiOH: XRD patterns of LiOH obtained via the hydrolysis process of lithium nitride and commercially available LiOH were analyzed and compared: The LiOH sample obtained through the process of hydrolysis and subsequent drying of Li_3N contains a minor quantity of LiCO3. In contrast, commercially available LiOH exhibits higher levels of lithium carbonate impurities. In addition, the lithium recovery rate achieved in the cycle process is an impressive 85%.

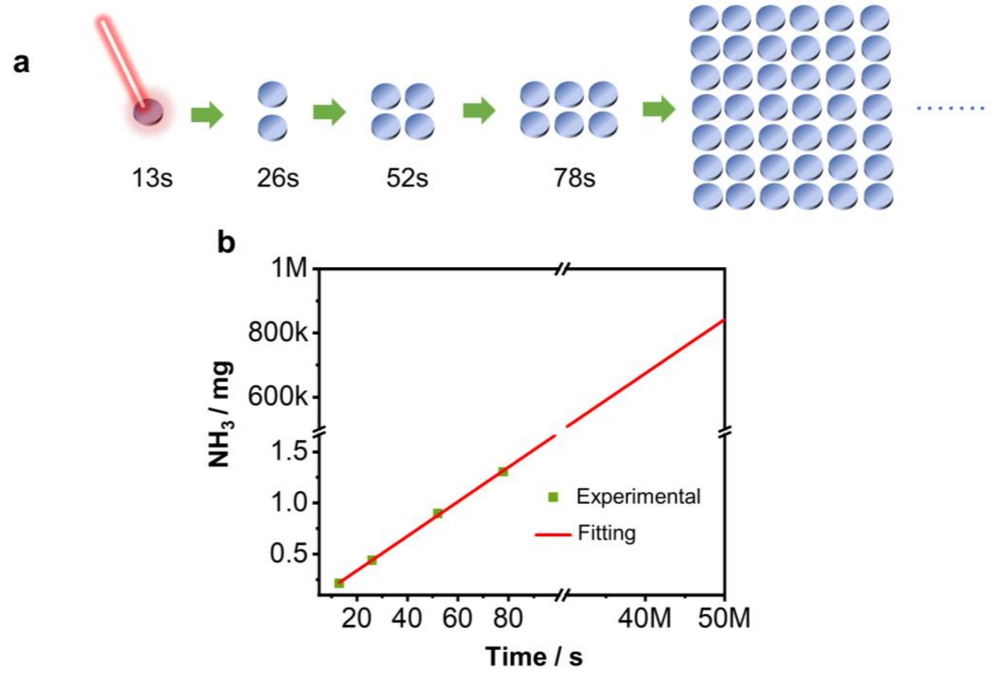

Supplementary Figure 33. Scale-up experiments: a) schematic diagram of prolonged scale-up experiments; b) The graph of the production of ammonia versus the irradiation time of the laser by using laser power of 152 kW cm^{-2} and scanning speed of 0.68 mm s^{-1} under ambient pressure.

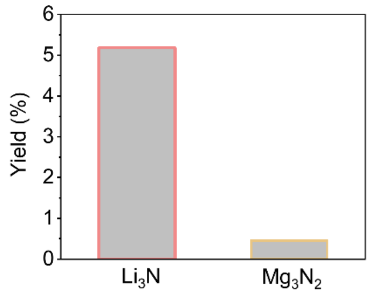

Supplementary Figure 34. Plot of the photons utilization efficiency for producing Li_3N and Mg_3N_2: the utilization rate of photons quantifies the efficiency by measuring the ratio between the number of photons necessary for ammonia production and the number of photons excited by the laser during a pulse period. The calculation of the theoretical photon requirement is based on the energy needed for metal-oxygen bond dissociation.

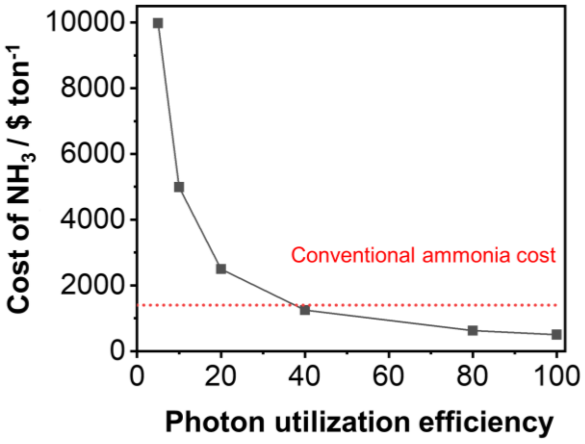

Supplementary Figure 35. Ammonia production cost analysis: the result was conducted for the LINF process, considering different photon utilization efficiencies. A comparison was made between the resulting ammonia costs in the LINF process and conventional industrial methods: In the article, we discussed the potential application of future solar-pumped laser converters. Therefore, the price per kilowatt-hour (kWh) mentioned here is derived from the current cost of solar photovoltaic power generation, which amounts to $0.031 per kWh (the price comes from *Wikipedia:https://en.wikipedia.org/wiki/Cost_of_electricity_by_source*). The average price of ammonia in 2022 is $1400 per ton. (the price comes from *"Farmdoc Daily"*: *https://farmdocdaily.illinois.edu/2022/09/fertilizer-prices-rates-and-costs-for-2023.html*)

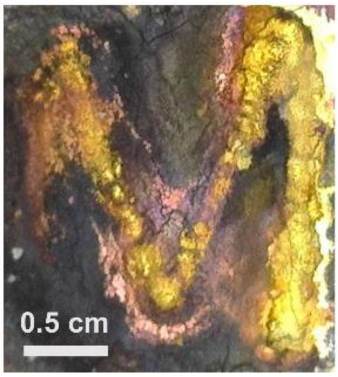

Supplementary Figure 36. A photograph of a laser-induced Li_3N film using an M-shaped pattern: after laser treatment, an M-shaped titanium nitride displayed as a golden yellow coating is obtained.

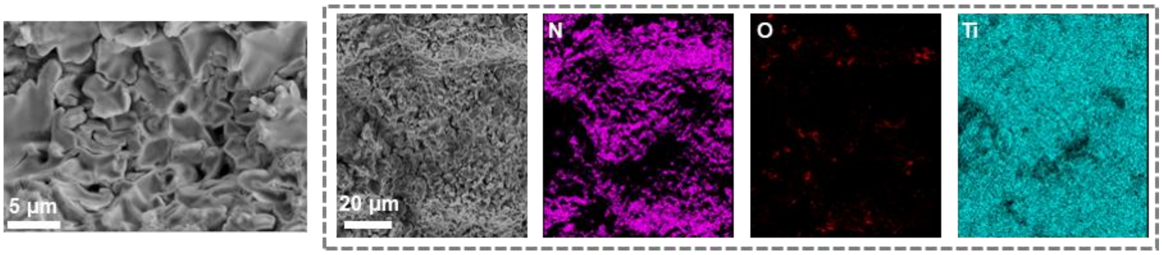

Supplementary Figure 37. SEM and elemental mapping analysis of TiN film: Top-view of SEM micrograph of a laser-induced TiN film with correlated EDX element maps.

Supplementary Table 1. Comparison with other reported methods.

Method	Selected work	Energy classification	Conditions	Yield rate μmol s^{-1}	Energy consumption kWh kg^{-1}	Yield %
Mechanochemical	*Nat. Nanotech.*, 2021, 16, 325	Mechanical	45 °C, 1 bar	0.3	1250	--
Haber-Bosch	*Nature*, 2020, 583, 391.	Thermal	400 °C, 1 bar	0.15	10833	--
Electrochemical	*Nature*, 2019, 570, 504; *Science*, 2021, 374, 1593; *Science*, 2021, 372, 1187; *Nature*, 2022, 609, 722.	Electrochemical	Room temperature, 15~20 bar	0.0002~0.15 μmol s^{-1} cm^{-2}	25~63333	--
Chemical looping	*Nat. Energy*, 2018, 3, 1067.	Thermal	Thermal	0.026	--	--
Photochemical	*ACS Energy Lett.*, 2019, 4, 1505.	Photochemical and thermal	Light, 1000 °C	55.6×10^{-6}	1056	--
Plasma	*Sci. Adv.*, 2019, 5, eaat5778	Plasma	Ambient temperature and pressure	7.19 ×10^{-3}	2270	--
Molten salt electrolysis	*Energy Environ. Sci.*, 2017, 10,1621	Electrochemical and thermal	450 °C, 1 bar for molten salt electrolysis, 25-100 °C for Li_3N synthesis	0.097 (22 °C, 0.5 h) 0.014 (22 °C, 12 h)*	--	--
**Laser**	**This work**	**Laser**	**25 °C, 1.0~7.5 bar**	**43.3 μmol s^{-1} cm^{-2} (Li_3N) / 94.11 μmol s^{-1} cm^{-2} (Li_3N+TiN)**	**322.7 (Li_3N) / 191.2 (Li_3N+TiN)**	11.2[£]

* The results incorporate the actual production of lithium under a current of 0.2 A for 1000 s. The calculation of the final ammonia yield takes into account the cumulative time of molten salt electrolysis, Li nitriding, and the conversion rate involved.

£ In the study, the highest ammonia yield corresponds to a Li_2O conversion of 11.2%. XRD and XPS analysis confirmed a reaction selectivity of nearly 100% for lithium oxide converting into lithium nitride, with no significant by-products detected. Consequently, the yield is approximately 11.2%.

Supplementary Table 2. List of bond dissociation energies of metal-oxygen bond

Bond	ΔHf_{298} (kJ/mol)
Li-O	341
Mg-O	394
Al-O	512
Ca-O	464
Zn-O	284

Source: J. A. Dean, "Properties of Atoms, Radicals, and Bonds" in *Lange's handbook of chemistry* (McGRAW-HILL, INC., 1999)

Note: It is essential to note that besides the breaking of metal-oxygen bonds plays a crucial role in the LINF process, the reactivity of zero-valent metals with nitrogen is another significant factor influencing nitride formation. Among these metals, lithium exhibits the highest level of reactivity and can even react with nitrogen at room temperature, leading to the formation of lithium nitride [28]. In contrast, the formation of zinc nitride necessitates the reaction of zinc with ammonia at temperatures exceeding 600 °C [29].

Supplementary Table 3. The content of N in TiN film was determined by ONH element analysis: synthesized TiN by using Li_2O as medium precursor and the corresponding amount of ammonia obtained after hydrolysis, compared with the ammonia generated by the hydrolysis of lithium nitride.

Samples			Atmosphere	TiN			Li_3N
Power (W)	Speed (mm s^{-1})	Pressure (Bar)	$^{14}N_2$/$^{15}N_2$	Weight of N (μg)	Weight of TiN (μg)	NH_3 form TiN (μg)	NH_3 form Li_3N (μg)
34.58	0.68	1	$^{14}N_2$	110	488	134	214
34.58	0.68	3	$^{14}N_2$	137	604	166	224
34.58	0.68	5	$^{14}N_2$	205	907	249	246
34.58	0.68	7.5	$^{14}N_2$	317	1403	385	268
34.58	0.68	1	$^{15}N_2$	130	576	158	216

Supplementary Table 4. Reaction energetics for Li_2O adatom defect on (111) and (211) surfaces

**Adatom defect Reaction**	**ΔE (eV) (111)**	**(211)**
$Li_2O \leftrightarrow Li_{2-x}OLi_{ad}^{+}$	1.82	1.25
$Li_{2-x}OLi_{ad}^{+} \leftrightarrow Li_{2-x}OLi_{ad}^{0}$	3.20	-
$Li_{2-x}OLi_{ad}^{0/+} + N_2 \leftrightarrow Li_{2-x}OLi_{ad}^{0/+}N_2$	-0.80	-0.88
**Overall reaction**		
$Li_2O + N_2 \leftrightarrow Li_{2-x}OLi_{ad}^{0/+}N_2$	4.22	0.37

Supplementary Table 5. Reaction energetics for Li_2O peroxide defect on (111) and (211) surfaces

**Peroxide defect Reaction**	**ΔE (eV) (111)**	**(211)**
$Li_2O \leftrightarrow Li_2O(O_2v)$	6.65	6.28
$Li_2O(O_2v) + N_2(g) \leftrightarrow Li_2O_{2-x}N_2$	-1.98	-1.77
**Overall reaction**		
$Li_2O + N_2(g) \leftrightarrow Li_2O_{2-x}N_2$	4.67	4.51

Supplementary Table 6. Reaction energetics for Li_2O anti-site defect on (111) and (211) surfaces

**Anti-site defect Reaction**	**ΔE (eV) (111)**	**(211)**
$Li_2O \leftrightarrow Li_2O(O_{2p})Li^0$	8.25	5.42
$Li_2O(O_{2p})Li^0 + N_2(g) \leftrightarrow Li_2O(O_{2p})Li^0N_2$	-1.54	-1.48
**Overall reaction**		
$Li_2O + N_2(g) \leftrightarrow Li_2O(O_{2p})Li^0N_2$	6.71	3.94

Supplementary Table 7. O-O bond distance in the Li_2O defected surfaces

	**O-O bond distance (Å)**	
**Li_2O defect**	**(111)**	**(211)**
peroxide	1.525	1.530
anti-site	1.488	1.523

Supplementary Table 8. N-N bond distance in the Li_2O defected surfaces

	**N-N bond distance (Å)**	
**Li_2O defect**	**(111)**	**(211)**
adatom	1.176	1.165
peroxide	1.294	1.283
anti-site	1.299	1.270

Supplementary Table 9. Reaction energetics for MgO adatom defect on (100) surface

**Adatom defect Reaction**	**ΔE (eV) (100)**
$MgO \leftrightarrow Mg_{1-x}OMg_{ad}$	6.22
$Mg_{1-x}OMg_{ad} + N_2(g) \leftrightarrow Mg_{1-x}OMg_{ad}N_2$	-0.37
**Overall reaction**	
$MgO + N_2(g) \leftrightarrow Mg_{1-x}OMg_{ad}N_2$	5.85

Supplementary Table 10. Reaction energetics for MgO peroxide defect on (100) surface

**Peroxide defect Reaction**	**ΔE (eV) (100)**
$MgO \leftrightarrow MgO(O_{2v})$	7.04
$MgO(O_2v) + N_2(g) \leftrightarrow MgO(O_{2v})N_2$	-0.69
**Overall reaction**	
$MgO + N_2(g) \leftrightarrow MgO(O_{2v})N_2$	6.36

Supplementary Table 11. Li_2O oxygen and lithium vacancy formation energies for (111) and (211) surfaces

Vacancy formation energies<br>Reaction	ΔE (eV)<br>(111)	 (211)
$Li_2O \leftrightarrow Li_2O_{1-x} + 0.5\ O_2(g)$	6.67	5.95
$Li_2O \leftrightarrow Li_2O_{1-2x} + O_2(g)$	13.43	11.06
$Li_2O \leftrightarrow Li_{2-x}O + Li(g)$	5.95	2.42

Supplementary Table 12. MgO oxygen and magnesium vacancy formation energies for (100) surface

Vacancy formation energies<br>Reaction	ΔE (eV)<br>(100)
$MgO \leftrightarrow MgO_{1-x} + 0.5\ O_2(g)$	6.32
$MgO \leftrightarrow MgO_{1-2x} + O_2(g)$	12.64
$MgO \leftrightarrow Mg_{1-x}O + Mg(g)$	10.20

Supplementary Table 13. Bulk formation free energetics for Li_2O and Li_3N

Reaction	ΔG (eV)
$2Li_2O\ (s) \rightarrow 4Li\ (s) + O_2(g)$	11.92
$6Li\ (s) + N_2\ (g) \rightarrow 2Li_3N\ (s)$	-4.07

Supplementary Table 14. Correlation between experiment time in LINF process and laser scanning speed

Laser Scanning Speed (mm s^{-1})	Experiment Time (s)
0.17	53
0.34	26
0.68	13
1.02	9
1.36	7

Supplementary References

1. Chen, G. *et al.* Saving the Energy Loss in Lithium-Mediated Nitrogen Fixation by Using a Highly Reactive Li_3N Intermediate for C−N Coupling Reactions. *Angew. Chemie Int. Ed.* **61**, (2022).

2. Kresse, G. & Hafner, J. Ab initio molecular dynamics for liquid metals. *Phys. Rev. B* **47**, 558–561 (1993).

3. Kresse, G. & Hafner, J. Ab initio molecular-dynamics simulation of the liquid-metal–amorphous-semiconductor transition in germanium. *Phys. Rev. B* **49**, 14251–14269 (1994).

4. Kresse, G. & Furthmüller, J. Efficient iterative schemes for ab initio total-energy calculations using a plane-wave basis set. *Phys. Rev. B* **54**, 11169–11186 (1996).

5. Kresse, G. & Furthmüller, J. Efficiency of ab-initio total energy calculations for metals and semiconductors using a plane-wave basis set. *Comput. Mater. Sci.* **6**, 15–50 (1996).

6. Perdew, J. P., Burke, K. & Ernzerhof, M. Generalized Gradient Approximation Made Simple. *Phys. Rev. Lett.* **77**, 3865–3868 (1996).

7. Blöchl, P. E. Projector augmented-wave method. *Phys. Rev. B* **50**, 17953–17979 (1994).

8. Kresse, G. & Joubert, D. From ultrasoft pseudopotentials to the projector augmented-wave method. *Phys. Rev. B* **59**, 1758–1775 (1998).

9. Almora-Barrios, N., Carchini, G., Błoński, P. & López, N. Costless Derivation of Dispersion Coefficients for Metal Surfaces. J Chem Theory Comput 10, 5002–5009 (2014).

10. Boes, J. R., Mamun, O., Winther, K. & Bligaard, T. Graph Theory Approach to High-Throughput Surface Adsorption Structure Generation. *The Journal of Physical Chemistry A* **123**, 2281–2285 (2019).

11. Monkhorst, H. J. & Pack, J. D. Special points for Brillouin-zone integrations. *Phys. Rev. B* **13**, 5188–5192 (1976).

12. Neugebauer, J. & Scheffler, M. Adsorbate-substrate and adsorbate-adsorbate interactions of Na and K adlayers on Al(111). *Phys. Rev. B* **46**, 16067–16080 (1992).

13. Togo, A., Chaput, L., Tadano, T. & Tanaka, I. Implementation strategies in phonopy and phono3py. *J. Phys.: Condens. Matter* **35**, 353001 (2023).

14. Togo, A. First-principles Phonon Calculations with Phonopy and Phono3py. *J. Phys. Soc. Jpn.* **92**, 012001 (2023).

15. Wang, V., Xu, N., Liu, J.-C., Tang, G. & Geng, W.-T. VASPKIT: A user-friendly interface facilitating high-throughput computing and analysis using VASP code. *Comput. Phys. Commun.* **267**, 108033 (2021).

16. Henkelman, G., Uberuaga, B. P. & Jónsson, H. A climbing image nudged elastic band method for finding saddle points and minimum energy paths. *J. Chem. Phys.* **113**, 9901–9904 (2000).

17. Smidstrup, S., Pedersen, A., Stokbro, K. & Jónsson, H. Improved initial guess for minimum energy path calculations. *The Journal of Chemical Physics* **140**, 214106–7 (2014).

18. Hugh O. Pierson. *Nitrides, Handbook of Refractory Carbides and Properties, Characteristics, Processing and Applications*. (William Andrew, 1996).

19. Wood, K. N. & Teeter, G. XPS on Li-Battery-Related Compounds: Analysis of Inorganic SEI Phases and a Methodology for Charge Correction. *ACS Appl. Energy Mater.* **1**, 4493–4504 (2018)

20. Jiang, G. *et al.* Solution-processed high-k magnesium oxide dielectrics for low-voltage oxide thin-film transistors. *Appl. Phys. Lett.* **109**, 183508 (2016).

21. Iatsunskyi, I. *et al.* Structural and XPS characterization of ALD Al_2O_3 coated porous silicon. *Vacuum* **113**, 52–58 (2015).

22. Erdem, B. *et al.* XPS and FTIR Surface Characterization of TiO_2 Particles Used in Polymer Encapsulation. *Langmuir* **17**, 2664–2669 (2001).

23. Greczynski, G. & Hultman, L. X-ray photoelectron spectroscopy: Towards reliable binding energy referencing. *Prog. Mater. Sci.* **107**, 100591 (2020).

24. Peng, X. D., Edwards, D. S. & Barteau, M. A. Reactions of O_2 and H_2O with magnesium nitride films. *Surf. Sci.* **195**, 103–114 (1988).

25. Maarouf, M., Haider, M. B., Drmosh, Q. A. & Mekki, M. B. X-Ray Photoelectron Spectroscopy Depth Profiling of As-Grown and Annealed Titanium Nitride Thin Films. *Crystals* **11**, 239 (2021).

26. Rosenberger, L., Baird, R., McCullen, E., Auner, G. & Shreve, G. XPS analysis of aluminum nitride films deposited by plasma source molecular beam epitaxy. *Surf. Interface Anal.* **40**, 1254–1261 (2008).

27. Höche, D., Blawert, C., Cavellier, M., Busardo, D. & Gloriant, T. Magnesium nitride phase formation by means of ion beam implantation technique. *Appl. Surf. Sci.* **257**, 5626–5633 (2011).

28. McEnaney, J. M. *et al.* Ammonia synthesis from N_2 and H_2O using a lithium cycling electrification strategy at atmospheric pressure. *Energy Environ. Sci.* **10**, 1621–1630 (2017).

29. Khan, W. S. & Cao, C. Synthesis, growth mechanism and optical characterization of zinc nitride hollow structures. *J. Cryst. Growth* **312**, 1838–1843 (2010).
