# Supplementary material for: Laser-induced nitrogen fixation
Source: Nat Commun. 2023 Sep 13;14:5668. doi: 10.1038/s41467-023-41441-0 (PMC10499830; doi:10.1038/s41467-023-41441-0)
Supplement: Supplementary file 3 — Description of Additional Supplementary Files [file 41467_2023_41441_MOESM3_ESM.pdf]

<u>Description of Additional Supplementary Files</u>

Supplementary Movie 1: LINF process of lithium oxide under nitrogen

Supplementary Movie 2: LINF process of lithium oxide under argon.

Supplementary Movie 3: LINF process of Magnesium oxide under nitrogen
